# Determination of Total Phenolics, Flavonoids, and Antioxidant Activity and GC-MS Analysis of Malaysian Stingless Bee Propolis Water Extracts

**DOI:** 10.1155/2021/3789351

**Published:** 2021-10-22

**Authors:** Sharifah Nur Amalina Syed Salleh, Nur Ayuni Mohd Hanapiah, Hafandi Ahmad, Wan Lutfi Wan Johari, Nurul Huda Osman, Mohd Razif Mamat

**Affiliations:** ^1^Faculty of Forestry and Environment, Universiti Putra Malaysia, 43400 UPM, Serdang, Selangor Darul Ehsan, Malaysia; ^2^Department of Veterinary Preclinical Sciences, Faculty of Veterinary Medicine, Universiti Putra Malaysia, 43400 UPM, Serdang, Selangor Darul Ehsan, Malaysia; ^3^Department of Physics, Faculty of Science, Universiti Putra Malaysia, 43400 UPM, Serdang, Selangor Darul Ehsan, Malaysia; ^4^Indo-Malayan Stingless Bee Repository, Malaysia Genome Institute, Jalan Bangi, 43000 Kajang, Selangor Darul Ehsan, Malaysia

## Abstract

Propolis contains polyphenolic compounds such as flavonoids and phenols that are able to demonstrate a broad spectrum of biological activities including antioxidant, antibacterial, and many more. This study was carried out to determine the total phenolics, flavonoids, and antioxidant activity of water-extracted propolis samples from three different Indo-Malayan stingless bee species, namely, *Tetrigona apicalis*, *Tetrigona binghami*, and *Homotrigona fimbriata*. Total phenolic and flavonoid contents were evaluated using Folin–Ciocalteu colorimetric and aluminium chloride methods, respectively, while the antioxidant activity was analysed using 1,1-diphenyl-2-picrylhydrazyl (DPPH) free radical scavenging assay. Results indicated that *H. fimbriata* extracts exhibit the highest TPC, TFC, and antiradical activity among all samples tested. Interestingly, the data also showed that the higher the concentration of the extract used, the higher the antioxidant activity exhibited by the samples. Statistically, there were no significant differences recorded between the different bee species' propolis studied. In conclusion, the propolis extracts showed stronger antioxidant potential with higher TPC and TFC values. This study also noted the presence of bioactive compounds from local stingless bee propolis that could potentially be utilised for their medicinal and health benefits.

## 1. Introduction

Propolis is generally made up of 50% resinous substances, 30% wax, 10% essential oils, 5% pollens, and 5% minor constituents such as impurities, amino acids, soils, and dead bees. The propolis extracts are known to contain numerous beneficial chemical compounds and biological activities that can be utilised in sectors such as pharmaceutical and health for their antimicrobial, antifungal, antiviral, anticancer, antidiabetic, antioxidant activities, and many more [1, 2]. It has been observed that honey bees mostly construct their nests or combs using waxes, unlike stingless bees, which often build their nests using plant resins collected or propolis. This is the reason why stingless bees had been reported to produce more propolis than any other bee species [3, 4]. The chemical content found in the stingless bee species depends heavily on factors such as propolis geographical location, bee species, seasonality, plants and flora available at the propolis collection sites, as well as bees' preference towards the floral and resin sources [5–7].

**Figure 1:**
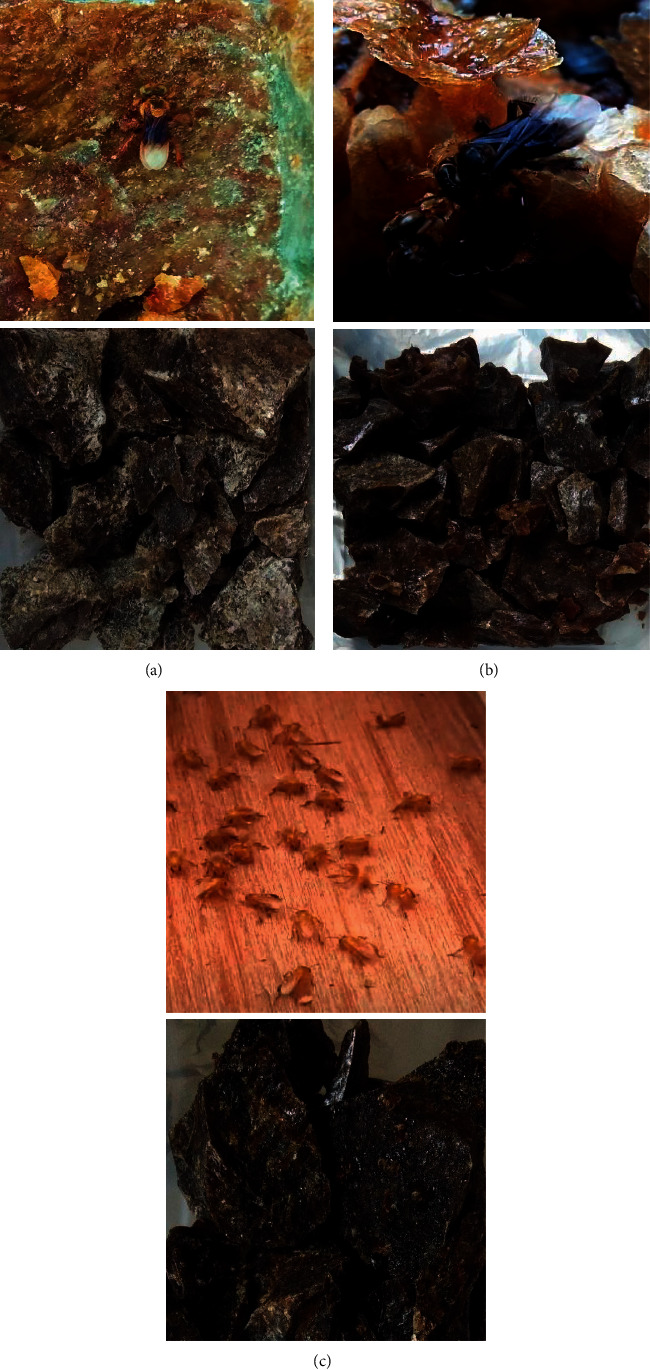
Images of stingless bees. (a) *Tetrigona apicalis*, (b) *Tetrigona binghami*, and (c) *Homotrigona fimbriata* captured along with their respective collected propolis.

It has been previously reported that there are about 29 species of stingless bees that can be found residing in the virgin forests in Malaysia, such as *Homotrigona fimbriata*, *Tetrigona apicalis*, and *Tetrigona binghami* (Figure 1) [8]. These bees are also among the common important meliponine species cultivated in Malaysia for pollinating and economic purposes. The bees used in this research are known to obtain resins from dipterocarps such as *Acacia* sp. for propolis construction.  Table 1 shows some attributes and characteristics of *H. fimbriata*, *T. apicalis*, and *T. binghami* used in present study [9–11].

These stingless bees also produce propolis extracts that can potentially be used as antioxidants, but more studies on the biochemical compounds and their biological activities are needed [11]. There are several factors that could influence the antioxidant activity of the propolis extracts, such as bee species, propolis collection site, solvents used in extraction, plant resinous source, and chemical compositions [12, 13]. The bioactive compounds present in the propolis are able to scavenge free radicals efficiently, which makes propolis a promising source of powerful natural antioxidants that can be utilised nowadays [14]. Antioxidants are important as the human mechanism of defence in fighting free radicals in the body, which makes the study on safe, consumable antioxidant activity of the propolis significant at the moment.

The medicinal and therapeutic properties of the stingless bee or meliponine propolis are also directly related to its chemical composition [15]. It has been proven that polyphenol components found in the propolis extracts play significant roles in exhibiting potent biological properties, such as antioxidant activity [16]. Studies conducted had shown that terpenoids and flavonoids are the two most commonly identified classes in the stingless bee propolis and flavonoids such as flavonols 3′-methyl quercetin, kaemferol-7-methyl ether, and flavone tricetin are compounds that had been most commonly found in the Brazilian meliponine propolis [16, 17].

Therefore, this study was carried out in order to investigate the total phenolic and flavonoid contents as well as antioxidant activity found in the Malaysian stingless bee propolis water extract since reports on the chemical content of the Malaysian meliponine propolis are very limited. In fact, the study on chemical compositions of the propolis extract is vitally important because propolis has been a subject of interest in the pharmaceutical and health sectors nowadays due to its potent chemical and biological properties. There had also been very few studies on the antiradical activity and biochemical contents of Malaysian stingless bee propolis using water as the extraction solvent.

It had been reported that the use of alcohol as the extraction solvent has several disadvantages including strong unpleasant taste and may cause some serious health issues when consumed for a long period of time. Besides, alcohol-extracted products are also inconsumable by individuals with alcohol intolerance [11]. Thus, propolis water extracts may potentially be a plausible alternative as they had been proven to be much safer, environmentally friendly, and biocompatible for consumption and utilisation in the cosmetic, food, and health sectors [18]. Hence, the chemical compounds and antioxidant activity of water-extracted local stingless bee propolis samples were investigated using a UV-VIS spectrophotometer. Besides, major chemical compounds in all of the propolis extracts were also identified using GC-MS.

## 2. Materials and Methods

### 2.1. Propolis Sample Collection

The stingless bee propolis samples used in the study were collected from Indo-Malayan Stingless Bee Repository at Malaysia Genome Institute, Selangor (N 2° 54′ 16.8732″ E 101° 46′ 5.61″), by scrapping the propolis toppings from *Tetrigona* sp. and *Homotrigona* sp. stingless bee hives [19]. The samples were chosen from three different stingless bee species, namely, *Tetrigona apicalis, Tetrigona binghami*, and *Homotrigona fimbriata* (Figure 1). Raw propolis samples were then kept in cool and dark places to prevent photodegradation prior to further investigation.

### 2.2. Propolis Water Extraction

The samples were extracted by using the maceration technique, as carried out in [20]. Approximately 40 g of each sample was crushed into small pieces and extracted with 100 mL of distilled water (2 : 5 m/v). Next, the samples were heated on a hot plate (Jenway 1000 Series, UK) with constant stirring at 70°C for 5 min. The samples were then placed at room temperature for a period of 24 hours in darkness before being filtered. The resulting solution was filtered using Whatman No 1 filter paper and concentrated under low temperature using freeze dry (Christ/RVC2-18/MZ2C) treatment. Finally, the extracted samples were kept at −20°C prior to sample analysis. The extracted samples were tagged as S1: *Tetrigona apicalis*, S2: *Tetrigona binghami*, and S3: *Homotrigona fimbriata*.

### 2.3. Determination of Total Phenolic Contents

The total phenolic content of each propolis extract was determined using the Folin–Ciocalteu colorimetric method as mentioned in [21] with slight modifications. 0.2 mL of each sample extract was placed into respective vials, and 0.8 mL of 10% Folin–Ciocalteu's reagent was then added to make up the final volume of 1 mL in each vial. After 5 min of incubation at room temperature, 1 mL of 8% sodium carbonate solution was added to the mixture and mixed with 95% ethanol until final volume in each vial up to 3 mL. The mixture was kept under the dark condition for 50 min, and the absorbance readings were measured at 725 nm using a UV-VIS spectrophotometer (Beckman Coulter/DU 730). Gallic acid (mg/mL GAE) was used as a standard solution to obtain the standard calibration curve.

### 2.4. Determination of Total Flavonoid Contents

Total flavonoid contents of the samples were determined using the aluminium chloride calorimetric method in [22] with slight modifications. Rutin was used as the standard and was prepared in concentrations range between 25 and 250 *μ*g/mL to obtain the standard calibration curve. Each extract and sample were then mixed with 0.5 mL aluminium chloride (10%) and 0.5 mL of 1 M potassium acetate. Next, the mixtures were allowed to stand for 30 min in the dark at room temperature and the absorbance was measured at 510 nm using a UV-VIS spectrophotometer (DU 730 Beckman Coulter). The absorbance measurements obtained were then compared with the standard calibration curve plotted to determine the flavonoid concentration in the samples. Total flavonoid content (TFC) of each sample was expressed in rutin equivalent (mg/mL RE). All of the steps taken were done in triplicate.

### 2.5. Determination of Antioxidant Activity

The antioxidant activity of each extracted sample was measured by using 2,2-diphenyl-1-picrylhydrazyl (DPPH) free radical (95%) scavenging assay (Alfa Aesar, Thermo Fisher Scientific, USA) as described in [13] with slight modifications. This reagent also serves as an antioxidant detector in which the purple colour of DPPH reagent will turn yellow or colourless based on the electron transfer or hydrogen donor of a compound. Methanol acts as a blank sample solution, and ascorbic acid (Sigma Aldrich, USA) is used as a positive control.

The DPPH stock solution was prepared by mixing 4 mg of DPPH powder in 100 mL of methanol and was left in the dark and cool condition until further analysis. Next, the sample stock solution was prepared at different concentrations (1, 2, 3, 4, and 5 mL) and methanol was added to get the total volume of 10 mL in each test tube. The stock solution was given a gentle mix, and 1 mL of each sample solution was transferred to volumetric flasks using a micropipette. Then, 3 mL of DPPH stock solution was placed, and methanol was added to get the final volume of 10 mL in each flask. The volumetric flasks containing the mixture were shaken vigorously and incubated at room temperature for 30 min under the dark condition. The absorbance was measured at a wavelength of 517 nm using a UV-VIS spectrophotometer (DU 730 Beckman Coulter). All of the steps were performed in triplicate. The ability of all sample extracts and positive control to scavenge the DPPH free radical was calculated using the following equation:

(1) $$\begin{array}{c} \text{DPPH scavenging activity }\left(\%\right)=\frac{A_{0}-A_{1}}{A_{0}}\times 100\%, \end{array}$$

where *A*_0_ represents the absorbance of the control sample and *A*_1_ represents the absorbance of sample extract with DPPH stock solution. Finally, the result of DPPH activity was expressed as IC_50_ values, which determined the concentration of the sample needed to inhibit 50% of DPPH free radical. The lower the IC_50_ value, the higher the antioxidant efficiency.

### 2.6. GC-MS Analysis for Bioactive Components

The chemical composition analysis was performed by using the gas chromatography mass spectrometry (Perkin Elmer Clarus 600 GCMS coupled to TurboMatrix Headspace Sampler 40) equipped with the National Institute of Standards and Technology (NIST) library data. A capillary column Elite 5MS (30m × 250 mm) of 0.25 *μ*m film thickness was used in this study. The acquisition parameters used were as follows: the column initial temperature was at 80°C (held for 4 min) and was increased to 250°C at 3°C/min and then to 300°C (held for 15 min) at 20°C/min; the injector was at a temperature of 250°C; and the source temperature at 280°C. The split ratio was 100 : 1, helium was the carrier gas, the flow rate was 1 mL/min, scan range was 35–500 Da, solvent delay time was 2 min, and the injected volume was 0.4 mL in each vial [23]. The compounds were selected based on the comparison from the National Institute of Standards and Technology (NIST) library. The compounds that showed 80% similarity with chemical compounds from NIST were selected for this study.

### 2.7. Statistical Analysis

Analyses were carried out in triplicate, and the results were expressed as means ± standard deviations. The Anderson–Darling normality test and one-way analysis of variance (ANOVA) were performed to determine the significant differences between TPC, TFC, and IC_50_ values among different stingless bee species by using Statistical Package for Social Sciences (IBM SPSS Statistics Software, 22.0 version). Differences were considered significant when *p* < 0.05. Other than that, Pearson's coefficient correlation was also used to evaluate the relationship between the antioxidant activity of each propolis extract with the concentration of total phenolic and flavonoid contents yield in each propolis extract.

## 3. Results and Discussion

**Figure 2:**
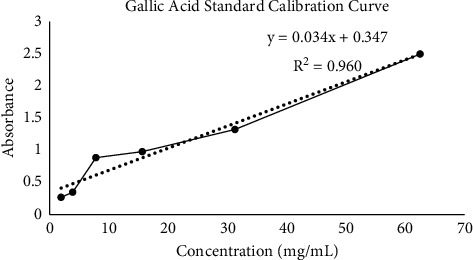
Standard calibration curve of gallic acid.

Propolis extracts are known to be rich with polyphenols, and therefore, the concentrations of total phenolics and flavonoids of water-extracted Malaysian stingless bee samples were analysed and determined in the present study.  Table 2 shows the total phenolic and flavonoid contents obtained from the chemical analysis of three stingless bee propolis water extracts investigated, while Figure 2 shows the standard curve plotted used to estimate phenolics (TPC) in the propolis extracts with regression equation *y* = 0.0344*x* + 0.347 and *r*^2^ = 0.9607. The concentrations obtained were expressed in gallic acid equivalence (mg/mL GAE).

It can be seen that TPC was the highest in *H. fimbriata* extracts with 13.21 mg/mL, followed by *T. binghami* and *T. apicalis* extracts with 10.11 and 7.60 mg/mL, respectively. The results obtained from this study also corroborated by a study [10] on Malaysian stingless bee ethanolic propolis extracts, showing *H. fimbriata* extracts to contain the highest amount of phenolics at 16.2 mg/mL, followed by *T. apicalis* with 13.9 mg/mL and *T. binghami* extracts at 5.7 mg/mL. Furthermore, investigation on propolis extracts of several Indonesian stingless bee species also revealed phenolic contents between 10 and 28.65 mg/mL [24]. Reports had shown that phenolic compounds found in propolis are able to help enhance human health and improve biological actions such as anticancer, anti-inflammatory, and antimicrobial activities effectively [25].

**Figure 3:**
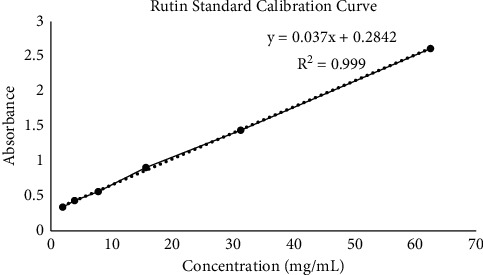
Standard calibration curve of rutin.

A standard calibration curve of rutin in Figure 3 was obtained from the absorbance of different concentrations of rutin (0–125 mg/mL). The total flavonoid content (TFC) of the extracts expressed in rutin equivalents (mg/mL RE) was calculated from the graph of absorbance against rutin concentration plotted, *y* = 0.0373*x* + 0.2842 with linearity *r*^2^ = 0.9994.

The highest TFC observed was from *H. fimbriata* propolis water extract, which was 34.53 mg/mL, while the lowest TFC recorded was from the extract of *T. binghami* species with 34.17 mg/mL. *T. apicalis* water extract exhibited a TFC mean value of 34.50 mg/mL. A research done on propolis extracts of both Mexican stingless and honey bees, *Melipona beecheii* and *Apis mellifera*, reported TFC values of 7.68 and 17.23 mg/g, respectively [26]. Other than that, Asem et al. [27] had also documented in their study that Malaysian stingless bee propolis extracts, including *T. apicalis*, contain flavonoids, which may play a role in propolis extracts exhibiting potent antioxidant activities. Differences in the TFC values shown by the propolis samples are also due to factors such as different stingless bee species, bee plant preferences, and extraction solvents used [28].

The DPPH, a free radical compound, was used to determine the scavenging ability of free radicals in different propolis samples by means of donating hydrogens through antioxidants [10]. The antioxidant activity was evaluated through the scavenging effect of DPPH on the extracts by measuring the DPPH inhibition percentage of each of the propolis extract. The concentrations of extract used were also varied in order to study the relationship between the extract volume used and its antioxidant effect.

Table 3 shows the percentage of DPPH scavenging activity and IC_50_ values of three different stingless bee propolis extracts at concentrations between 1 and 5 mg/mL. The results demonstrated that the percentage of DPPH scavenging activity of *H. fimbriata* (56.91%) particularly at the concentration of 5 mL was higher than ascorbic acid (48.22%), *T. apicalis* (47.56%), and *T. binghami* (41.87%). Besides, the IC_50_ value of *H. fimbriata* (3.95 mg/mL) was also lower than that of ascorbic acid (5.08 mg/mL), *T. apicalis* (5.18 mg/mL), and *T. binghami* (6.14 mg/mL) with good correlation coefficient, *r*^2^ = 0.987.

When comparing the outcomes of the present study to those reported by Rosli et al. [29], it has been proven that *Trigona* sp. is potentially capable of demonstrating great antioxidant activity. Moreover, the IC_50_ inhibition value for the DPPH assay in the study was recorded at 4.27 mg/mL with correlation coefficient *r*^2^ = 0.9461. However, in another previous study [30], it had been documented that the *T. apicalis* propolis ethanolic extract exhibited the weakest antioxidant capacity among other species with lesser flavonoid and phenolic compounds detected in the extract. The inconsistency of the antioxidant activity and different amounts of chemical compositions displayed might be due to botanical sources, geographical zones, and types of solvent used during propolis extraction [31]. It can also be seen on average that the lower the concentration of propolis extract used, the lower the antioxidant effect exhibited by the extracts. This showed that propolis concentration and solvent volume used play an important role in producing antioxidant properties of the propolis extracts.

Table 4 reveals that there are strong correlations between the total flavonoid and phenolic contents of the propolis extracts with antioxidant activities. The data obtained on Pearson's correlation coefficient of TPC and TFC with antioxidant activity showed strong positive correlations with *r* = 0.662 and *r* = 0.832, respectively.

These results indicated that the higher the flavonoids and phenolics found in propolis extracts, the higher the antioxidant activity displayed by the extracts. This finding is also agreeable with those reported by Pobiega et al. [18], which showed a strong positive correlation between total phenolic and flavonoid contents with the antioxidant activities exhibited by propolis samples studied. However, there is no correlation determined between the TPC and TFC of the stingless bee propolis extracts tested with *r* = 0.135.

**Figure 4:**
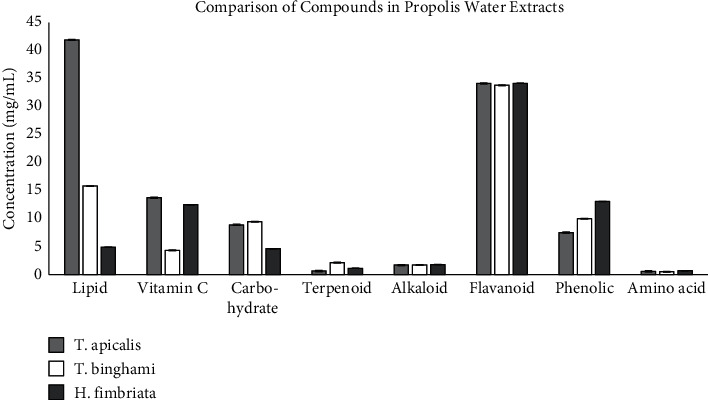
Comparison of detected compounds from GC-MS screening of *T. apicalis*, *T. binghami*, and *H. fimbriata* propolis water extracts.

**Figure 5:**
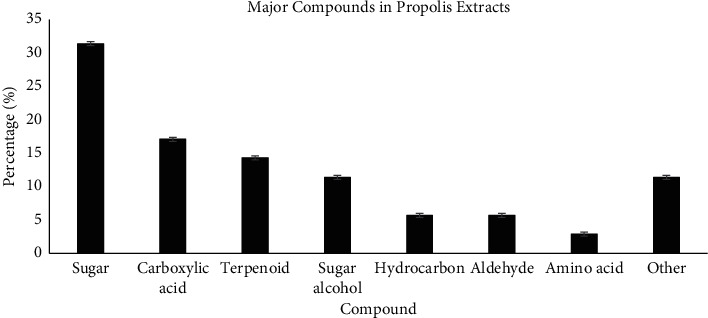
Major chemical compounds detected in GC-MS analysis of *T. apicalis*, *T. binghami*, and *H. fimbriata* propolis water extracts.

Propolis contains various bioactive compounds that may differ according to places of origin or collection sites [12]. It had been reported that resins collected for propolis are usually plant species-specific and the chemical compounds may differ greatly between and within the same plant families, including among closely related ones [32].  Table 5 presents major compounds detected in all stingless bee propolis water extracts tested using GC-MS analysis as shown in Figure 4. A total of 35 different chemical compounds had been identified and listed. It can be seen that the Malaysian stingless bee propolis water extracts are characterised by groups such as sugars (31.4%), carboxylic acids (17.1%), terpenoids (14.3%), sugar alcohols (11.4%), hydrocarbons (5.7%), aldehydes (5.7%), amino acid (2.9%), and other compounds (11.4%) as shown in Figure 5.

Reducing sugars such as ribose, fructose, glucose, and galactose made up 31.4% of total compounds identified in the extracts. *T. apicalis* extracts produced the highest amount of sugars, followed by *T. binghami* and *H. fimbriata* extracts, as shown in Table 4. Most studies on sugars had been carried out on stingless bee honeys. A study conducted on Malaysian *H. itama* honey revealed that it contains high levels of glucose and fructose that could be due to the bee floral source [33]. Moreover, it had also been reported in [18] that water extracts usually able to yield more carbohydrates, terpenes, and other nonphenolic compounds compared with alcoholic extracts, and this could also be one of the contributing factors to the high percentage of sugars detected in the studied samples when analysed.

Next, carboxylic acids such as propanoic and octadecatrienoic acids are some compounds that can be found in *H. fimbriata* and *T. apicalis* extracts. No carboxylic acids had been identified in *T. binghami* extract. Tricosadiynoic acid identified in *H. fimbriata* extract had also been reported to be able to treat high-fat diet and obesity-related diseases by reducing the insulin level and body weight gain in rats studied [34, 35].

Other than that, terpenoids are also an important group detected in the samples for their biological activities and aromatic qualities [36]. *H. fimbriata* extract contains the highest amount of terpenoids followed by *T. apicalis* and *T. binghami* extracts. Some terpenoids identified in the samples are phorbol, isolongifolol, germacrene D, isoaromadendrene epoxide, and *α*-eudesmol. Research on Brazilian honey and stingless bee propolis extracts revealed that components such as germacrene D can only be detected in stingless bee propolis and *α*-eudesmol found in propolis volatile oils contains antibacterial properties [37]. However, there are very few studies regarding stingless bee propolis volatiles compared with honey bees [38]. Phorbol had also been successfully identified in Indian propolis extract for the first time in 2015 [36].

Moreover, sugar alcohols such as ribitol, arabitol, arabinitol, and D-glucitol had also been discovered in all samples, with the highest amount detected in *T. binghami* extracts, followed by *T. apicalis* and *H. fimbriata* extracts. Sugar alcohols had also been documented in Brazilian *Melipona* sp. and *Tetragonisca angustula* propolis extracts [38]. However, hydrocarbons such as silane and butane were only identified in *T. binghami* extracts. Silane in propolis extracts can be used for wood protection effectively as biofriendly hydrophobic agents. Butane had also been identified in Polish propolis extracts in very small amounts [39, 40]. Furthermore, other constituents such as carboxaldehydes and amino acids had also been detected in *T. binghami* and *T. apicalis* extracts, respectively, in small amount. Pyroglutamic acid had been reported in Polish propolis recently. It had also been noted that amino acids discovered in propolis extracts were in very low amount [41].

It has been established that water is one of the safest, nontoxic solvents that can be used when extracting propolis [42]. According to [24], ethanol may be able to produce more flavonoids and phenolics in the extracts, which contribute to the antioxidant activity of the propolis as they are able to neutralise free radicals more efficiently, while water extracts usually produce more carbohydrates, terpenes, and other nonphenolic compounds. However, alcoholic extracts can have limited applications in the food and health industries. Although ethanolic extracts are commonly used in propolis sample preparation, they are known to produce pungent and unappetising taste, which could be disadvantageous to be used when administered orally in food and pharmaceutical sectors [43]. Water extracts have been proven to be a much safer and biocompatible option compared with alcoholic extracts; however, more research is needed in order to determine better nonalcoholic alternatives that can be used as extraction solvents to enhance the antioxidant capacity and solubility of the flavonoids in the propolis extracts.

## 4. Conclusion

This study found that propolis from Malaysian stingless bee species *T. apicalis*, *T. binghami*, and *H. fimbriata* are able to produce biochemicals that are potentially important in the pharmaceutical and health sectors. The concentration of TPC found in the propolis samples analysed is in the order of *H. fimbriata* > *T. binghami* > *T. apicalis*, while TFC and DPPH scavenging activity of the samples are in the order of *H. fimbriata* > *T. apicalis* > *T. binghami*. The data obtained from this study may hopefully be able to shed some light on the antioxidant activity and total flavonoids produced by the local stingless bee propolis through the water extraction method. Further investigations are needed in order to enhance the production of antioxidants and flavonoids in nonalcoholic propolis extracts so that it can be widely used in the food and medical fields in the near future.

## Figures and Tables

**Table 1:** General physical characteristics and propolis attributes of *H. fimbriata*, *T. apicalis*, and *T. binghami*.

Characteristics	Species		
	*H. fimbriata*	*T. apicalis*	*T. binghami*
Body length	8.10–8.29 mm	6.82–7.30 mm	6.61–7.75 mm
Body colour	Brown	Blackish brown	Blackish brown
Wing colour	Slight brownish base, semiclear apex	1/2 dark base, 1/2 transparent-tipped	1/4 dark base, 3/4 white-tipped
Propolis colour	Dark brown	Dark brown	Light brown
Propolis type	Hard and brittle	Hard and brittle	Hard and brittle

**Table 2:** Total phenolic content (TPC) and total flavonoid content (TFC) of *H. fimbriata*, *T. apicalis*, and *T. binghami* propolis water extracts (mg/mL).

Propolis	Total phenolic content	Total flavonoid content
*H. fimbriata*	13.21 ± 0.26	34.53 ± 0.11
*T. apicalis*	7.60 ± 0.13	34.50 ± 0.24
*T. binghami*	10.11 ± 0.19	34.17 ± 0.25

No significant difference detected for both TPC and TFC since *p* > 0.05.

**Table 3:** Percentage of DPPH scavenging activity and IC_50_ value of propolis water extracts.

Propolis	Concentration of sample (mg/mL)	Absorbance	DPPH scavenging activity (%)	IC_50_ (mg/mL)
*T. apicalis*	1	0.391	20.53	5.18
	2	0.364	26.02	
	3	0.315	35.98	
	4	0.282	42.68	
	5	0.258	47.56	
*T. binghami*	1	0.403	18.09	6.14
	2	0.356	22.64	
	3	0.323	34.35	
	4	0.312	36.59	
	5	0.286	41.87	
*H. fimbriata*	1	0.343	30.28	3.95
	2	0.313	36.38	
	3	0.274	46.31	
	4	0.245	50.20	
	5	0.212	56.91	
Ascorbic acid	1	0.355	19.17	5.08
	2	0.333	24.25	
	3	0.275	37.19	
	4	0.245	42.13	
	5	0.227	48.22	
	Blank	0.439	0.00	

**Table 4:** Pearson's correlation coefficient of TPC, TFC, and antioxidant activity of stingless bee propolis water extracts.

Assay	TPC	TFC	DPPH
TPC	1	0.135	0.662^*∗*^
TFC	0.135	1	0.832^*∗*^
DPPH	0.662^*∗*^	0.832^*∗*^	1

^*∗*^Correlation is significant at *p* ≤ 0.05.

**Table 5:** Major compounds detected in stingless bee propolis water extracts using GC-MS analysis.

Compound	Propolis		
	*H. fimbriata*	*T. apicalis*	*T. binghami*
*Terpenoid*			
Germacrene D	+	−	−
Isolongifolol	+	−	−
Phorbol	+	−	−
*α*-Eudesmol	−	+	−
Isoaromadendrene epoxide	−	−	+

*Sugar*			
D-Fructose	+	+	+
d-Galactose	+	+	+
*α*-D-Glucopyranoside	+	−	−
*α*-D-Galactopyranoside	+	−	−
d-Ribose	−	+	−
d-Glucose	−	+	−
D-Glucose	−	+	+
*α*-D-Mannopyranoside	−	+	−
Glycoside	−	+	−
Hexopyranose	−	+	+
*α*-D-Glucofuranoside	−	−	+

*Carboxylic acid*			
*α*-Linolenic acid	+	−	−
Tricosadiynoic acid	+	−	−
Octadecatrienoic acid	+	−	−
Butanedioic acid	−	+	−
Propanoic acid	−	+	−
Cyclohexene	−	+	−

*Sugar alcohol*			
Arabinitol	+	+	+
Ribitol	+	+	+
Arabitol	−	+	+
D-Glucitol	−	−	+

*Hydrocarbon*			
Silane	−	−	+
Butane	−	−	+

*Aldehyde*			
Dioxolane-4-carboxaldehyde	−	−	+
Cyclohex-1-en-1-carboxaldehyde	−	−	+

*Amino acid*			
Pyroglutamic acid	−	+	−

*Others*			
Trimethylsilyl ether of glycerol	+	+	+
3,8-Dioxa-2,9-disiladecane	+		+
Silanol	−	+	−
2-Mono-isobutyrin	−	−	+

## Data Availability

No data were used to support this study.
